# Research on Teleoperated Virtual Reality Human–Robot Five-Dimensional Collaboration System

**DOI:** 10.3390/biomimetics8080605

**Published:** 2023-12-13

**Authors:** Qinglei Zhang, Qinghao Liu, Jianguo Duan, Jiyun Qin

**Affiliations:** 1 China Institute of FTZ Supply Chain, Shanghai Maritime University, Shanghai 201306, Chinajyqin@shmtu.edu.cn (J.Q.); 2 Logistics Engineering College, Shanghai Maritime University, Shanghai 201306, China

**Keywords:** teleoperation, virtual reality, human–robot collaboration, visualization interface

## Abstract

In the realm of industrial robotics, there is a growing challenge in simplifying human–robot collaboration (HRC), particularly in complex settings. The demand for more intuitive teleoperation systems is on the rise. However, optimizing robot control interfaces and streamlining teleoperation remains a formidable task due to the need for operators to possess specialized knowledge and the limitations of traditional methods regarding operational space and time constraints. This study addresses these issues by introducing a virtual reality (VR) HRC system with five-dimensional capabilities. Key advantages of our approach include: (1) real-time observation of robot work, whereby operators can seamlessly monitor the robot’s real-time work environment and motion during teleoperation; (2) leveraging VR device capabilities, whereby the strengths of VR devices are harnessed to simplify robot motion control, significantly reducing the learning time for operators; and (3) adaptability across platforms and environments: our system effortlessly adapts to various platforms and working conditions, ensuring versatility across different terminals and scenarios. This system represents a significant advancement in addressing the challenges of HRC, offering improved teleoperation, simplified control, and enhanced accessibility, particularly for operators with limited prior exposure to robot operation. It elevates the overall HRC experience in complex scenarios.

## 1. Introduction

Industrial robots have become increasingly popular in modern manufacturing. In recent years, research and applications have demonstrated the pivotal role of robots, particularly in industrial settings such as assembly on workshop production lines, component repair in the aviation industry, material sorting, and packaging in manufacturing. These applications often involve breaking tasks into multiple work segments. Under the guidance of experienced workers, robots can effectively leverage their capabilities to execute desired operations or assist in completing specific industrial workflows. This process is commonly referred to as human–robot collaboration (HRC). In the HRC investigation in this study, the focus is on remote human operation of robots to accomplish tasks. However, HRC faces several challenges and issues. When humans and robots work in close proximity, they often struggle to meet safety requirements in industrial manufacturing. There is also a heightened demand for the experience and skills of operators, as well as the synchronization of actions in both time and space. Given that robots possess high precision in motion and humans exhibit adaptive intelligence, leveraging the strengths of both entities can significantly benefit industrial manufacturing and the application of industrial robots.

In this context, the development of teleoperation robot technology provides solutions to some of the challenges in HRC. Teleoperation robot technology involves controlling robots by collecting human body information to execute tasks. Information collected includes brainwave signals [1], muscle signals [2], human posture [3] and gestures, speech [4], location, and image data [5]. This information is gathered and processed using specific devices. It is then transmitted to local or remote computers through specialized communication methods and protocols, subsequently utilized by the robot’s operating system to perform tasks.

Teleoperation [6] differs from traditional methods like teaching and Programmable Logic Controller (PLC) control in that it enables remote control of robots. This approach satisfactorily meets the safety requirements in traditional industrial HRC, ensuring user safety. Moreover, remote operation allows industrial robots to effectively perceive the spatial position, posture, and actions of operators, among other information. The development of teleoperation technology is particularly meaningful for robots executing complex and labor-intensive tasks, especially those in extremely harsh environmental conditions. After years of extensive research, teleoperation robot technology has proven its effectiveness in a wide range of multidimensional applications. In these applications, operators only need to provide their skills remotely, eliminating the need for physical presence. This makes teleoperation deployment much safer in extremely hazardous and challenging environments than sending human personnel. Furthermore, it enhances the scientific and practical aspects of these operations. For instance, in NASA’s teleoperated robotic astronaut project [7], a remote-controlled robot was used to perform maintenance tasks on a space station. Following the Fukushima Daiichi nuclear disaster in 2011, the environment inside the nuclear power plant became exceptionally dangerous. The cost and risk of sending workers to the site for manual intervention were prohibitively high. Therefore, the deployment of several remote-controlled robots [8] was considered for task execution. Teleoperation not only excels in hazardous and complex environments, but also reduces delays caused by operators or experts who are not physically present and need to spend time on transportation. Remote surgery, for instance, has had a significant impact on the health care sector [9]. It allows for long-distance scenarios in which workers and experts can collaborate, reducing time spent on travel.

Current HRC interfaces primarily involve workers interacting with robots through 2D screen teaching pendants, control panels, and teaching keys, often requiring a certain level of experience to operate them proficiently. Gaining this experience necessitates extensive training and prolonged usage, enabling less experienced workers or newcomers to understand the principles of interaction and the methods for executing corresponding operations. In recent years of research, the combination of various intelligent devices has been proposed to achieve more intuitive visualization, remote control, and collaborative work. Emerging interactive devices and display terminals such as tablets, virtual reality (VR) [10,11], augmented reality (AR) devices [12], sound [13], smartphones, and gestures [14] have been integrated. These devices have brought new solutions to achieve a more intuitive visualization of teleoperation issues. Additionally, in advanced manufacturing, including digital twins [15], data analysis, and industrial big data, there is a demand for autonomy separate from the interface. Therefore, advanced HRC necessitates novel HRC to facilitate intuitive visualization, control, and autonomous collaboration. In these studies, VR devices simulate the real-world environment through computer technology, providing a remote operation and immersive experience. This offers new solutions for more intuitive visualization of teleoperation challenges.

In the present era, human interaction methods [16] have undergone a transformation, transitioning from traditional 2D interactions to more efficient and intuitively immersive 3D interaction approaches. In light of this evolution, VR technology has assumed a significant role in next-generation intelligent HRC [17,18,19,20,21]. VR has the capacity to create fully immersive virtual spaces, accessible only through VR devices, offering an enhanced, intuitive visualization of the created virtual environments. This extends to various contexts, including industrial scenarios and industrial equipment. Consequently, the synergy between VR and robotics presents vast research opportunities and utility. Nevertheless, it was only seven years ago that many developers began utilizing commercial VR devices. Since then, due to their support for novel HRC domains and the provision of a robust platform, research in related fields has rapidly advanced. Currently, most research and development on the application of VR in robotics predominantly focuses on the development of VR interfaces for robot operation. Over the past few years, research and development [22] related to VR in HRC can be categorized into five main dimensions:

(1) Control: emphasis is placed on linking operator commands or gestures with robot actions, facilitating the execution of tasks by the robot.
(2) Visualization: focusing on displaying information about the robot’s movements or various data to operators.
(3) Interaction: developing new interaction techniques or evaluating the best interactive experiences.
(4) Usability: simplifying operational complexity and barriers to enhance the user experience of VR interfaces.
(5) Infrastructure: primarily consists of system frameworks, application software, and plugins supporting the connection of VR devices with actual robots for interface development.

Throughout both research and practical applications, numerous cases involve user interactions with simulated robotic entities in virtual environments. These cases encompass remote operating interfaces, manufacturing assembly process simulations, HRC simulations, medical robot surgical practice, and more. All of these tasks can be executed within VR’s virtual environments, intuitively showcased on display platforms, delivering safer, cost-effective HRC experiences. VR’s prominence in the five dimensions of industrial integration makes it an attractive choice for interacting with simulated robots, providing a more immersive experience by better replicating real-world environmental visual cues.

Despite considerable achievements in teleoperating robots in VR, there remain unresolved challenges. The majority of research tends to focus on one or two dimensions out of the five, failing to simultaneously address the demands of safety, efficiency, intuitive visualization in teleoperation, and reducing operational complexity.

Current research on VR-based HRC faces challenges such as limited adaptability across multiple scenarios, performing relatively simplistic tasks, insufficient human–robot interaction, and inadequate visualization of information. Hence, we propose a novel Five-Dimensional Human–Robot VR Teleoperation System. This system can operate actual robots in conditions where human operators have little to no experience and are remote from the robot. The system integrates control, visualization, interaction, usability, and infrastructure dimensions to perform practical robotic operations. The robot’s control and infrastructure dimensions address the needs of remote control in teleoperation and reduce operational complexity. Additionally, this system can be adapted to a wide range of scenarios and devices, offering versatility. The combination of visualization, interaction, and usability dimensions meets the requirements of safety and intuitive visualization in HRC, providing an immersive experience. The contributions of this paper are as follows. This system leverages VR hardware interfaces to construct a virtual collaboration system where humans and robots collaborate. In this system, robots, with their high motion precision and physical limitations, track operator motion trajectories to complete assembly tasks. Robot movement is entirely controlled by operators, who possess adaptive intelligence, minimizing the impact of changing working environments and unpredictable factors. The proposed Five-Dimensional Human–Robot VR Collaboration System combines the strengths of humans (adaptive intelligence) and robots (high motion precision and physical limitations), effectively leveraging the advantages of VR devices in HRC. This VR-based HRC framework can be extended to other skilled manufacturing processes, such as welding, spraying, and painting.

Section 2 discusses recent research related to VR in HRC, Section 3 presents the system’s design and framework, Section 4 elaborates on the principles of the five-dimensional integration for assembly operation experiments, and Section 5 analyzes the results. Finally, Section 6 concludes the paper and outlines future work.

## 2. Related Work

### 2.1. Robot Teleoperation

Robot teleoperation involves the interaction between an operator who issues control commands, tactile interfaces or master devices, communication channels, and a remote robot [23]. In many studies related to HRC, operators manipulate robots using human movements or behaviors, often achieved through hand gestures, voice commands, or body postures. As a result, this research primarily relies on non-contact technologies, including wearable devices (controllers and display headsets) [24] and spatial tracking techniques (positioning and scene construction).

With recent advancements in communication technologies like 5G and Wi-Fi 6, coupled with the development of the tactile internet, it is now possible to control and monitor remote robots in real-time using mixed reality [25], all within a low-latency, high-precision communication environment [26]. The integration of the tactile internet with communication technologies has addressed stability and transparency issues that were previously hindered by network problems. Although there is relatively limited research concerning robots in this field [27], it holds immense potential for growth.

The aforementioned research shares similarities with our study in terms of creating virtual models corresponding to real-world scenarios, developing HRC interfaces, and enabling remote control of robots. However, what sets our study apart from these previous works is that it does not focus solely on the development of individual control or visualization aspects. Instead, it combines the advantages of these features, creating a seamless loop from human to VR devices, computer systems, and robots. This approach can adapt to various complex control scenarios, offering a simplified control system and a more intuitive visual interface, among other benefits.

### 2.2. Middleware and Data-to-Communication Protocol

In robot teleoperation technology, it is essential to collect data from physical terminal devices and sensors simultaneously and process and communicate these data between the operator, the computer, and the actual robot devices. This process is complex, and the data-processing formats used on different platforms and terminals can vary significantly. Integrating different devices from various vendors seamlessly is challenging. Therefore, an intermediary platform is needed to bridge different communication protocols and standardize data from various terminals, creating a smooth connection throughout the entire system. This is also a key goal of smart manufacturing [28]. Communication between robots typically relies on middleware such as the Robot Operating System (ROS), Open Platform Communications Unified Architecture (OPC UA), Ethernet/IP, Message Queuing Telemetry Transport (MQTT), and Data Distribution Service (DDS) [29]. In essence, the communication process involves data publishers who send data and data subscribers who receive the data. The connection between data from different terminals is managed by a central agent (e.g., MQTT or ROS) or is inherent in the data itself, including node addresses (e.g., DDS or OPC UA). With the support of these communication protocols, it becomes possible to connect real physical assets and fieldbuses from different vendors, enabling the flexible use of the entire manufacturing system without being constrained by incompatibility between platforms. For example, several studies have demonstrated the application of ROS in smart manufacturing. ROS is an open-source and widely recognized cloud robotics framework [30,31]. The various robot modules, sensor modules, communication modules, and application modules provided by the ROS platform make it convenient to build robot systems. Communication within the ROS is accomplished through topic subscription and publication, facilitating the deployment of various functional packages and reducing the computational burden through distributed processing methods, including inverse kinematics solving, image processing, and motion planning algorithms. For instance, ROS, in combination with the Unity game engine (version 2019.3.2f1), has been used for monitoring the robot welding process [32] and for simulation-based educational purposes [33]. ROS, along with lightweight virtualization technology, has also been employed to build Information Physical Systems for Automated Guided Vehicles (AGVs) [34]. Therefore, by combining data communication protocols with middleware, a versatile smart manufacturing platform can be constructed. Many robotics research groups have chosen ROS as their software of choice [35,36]. In the ROS platform, implementing teleoperation typically requires the use of ROS’s built-in visualization software, RViz, and the ROS Interactive Markers (IM) stack. RViz utilizes a graphical user interface displayed on a computer monitor with the support of the IM stack. However, compared to contemporary interaction interfaces, this interface is both complex and slow, lacking efficiency. Moreover, ROS platform requirements on the operator’s computer system can be high, resulting in a steep learning curve. Therefore, in this study, we build upon ROS by integrating Unity engine, which provides a simpler, more efficient, and visually intuitive control interface within the ROS platform. This integration allows for the transformation and communication of different data types between the VR interface’s three-dimensional visual rendering and HRC. Through the Unity platform, data from multiple terminals can be processed and operated upon, enabling seamless communication among the various modules of the system.

### 2.3. VR Robotics Interface

Robots are typically operated through open-source software within the ROS, such as RViz, MoveIt, and Gazebo, alongside specialized software like RoboDK and Siemens NX. These applications provide a wide range of functionalities, including robot control, offline simulation, deployment with superimposed images, path planning, and more. However, this software operates within a 2D computer interface, where users perform actions through clicks and commands, lacking the ability to perceive beyond the control instructions they provide. Enhancing the interaction platform between operators and robots to be more multidimensional would significantly improve the naturalness and intelligence of operations. In this study, we leverage VR devices to enhance human intuition and interaction with virtual entities, enabling interactions and control operations with real-world devices. Some research has demonstrated innovative interfaces for HRC [37], rendering virtual robot systems based on received joint angles from the actual robot’s controller. This increases the resemblance between virtual and actual robots. Operators can then intuitively manipulate the virtual robot using methods such as dragging the end-effector, VR controllers, or selecting options from 3D menus. These interactions are informed by the visual feedback of the actual robot’s current pose, making it possible to control the virtual robot by issuing commands while visually observing its motion. Such an approach revisualizes the robot’s movements more intuitively, allowing control of the real robot through feedback and control of the virtual robot’s motion. This approach effectively utilizes Unity engine and VR headsets to create an immersive virtual environment, constructing application scenarios for virtual robots that interact with real physical devices, such as the motion of actual robots, camera feeds, and sensor information. These are dynamically mapped to the user’s control interface and virtual environment. Other studies combine VR interfaces with haptic devices to provide an enhanced environment [17]. For example, Wang et al. [38] introduced a remote robot welding system using VR headsets as interactive devices. Operators control the movement of a welding gun mounted on the robot through programming while simultaneously observing camera feeds and welding parameters displayed on the robot’s VR model, improving welding control precision and keeping workers away from potentially hazardous welding scenes. The aforementioned studies demonstrate the attempts of experts and researchers to make robot motion, task execution, or production processes more intuitively accessible using VR. Building upon this previous research, the motivation of this study is to devise a method for presenting the status information of physical systems in a virtual environment on the internet in the most intuitive manner possible. The goal is to provide information feedback within a virtual environment that influences physical systems while reducing the learning curve for operators. Additionally, we aim to maximize human intelligence in combination with multiple terminals and devices.

In summary, this study primarily proposes an advanced data communication and system design framework and a human–robot application interface to fully exploit the advantages of VR technology in the field of robotics. This approach maximizes the integration of high-precision robot motion with human adaptive intelligence, distinguishing it from previous VR-based robot interface research. Our system operates within the Unity engine and ROS, where the robot’s motion information is visually presented, displaying specific coordinates of its movement. The diversified visual representation of motion information across various platforms and interfaces distinguishes it from traditional teach pendants, which typically show joint angles only. This enhances the precision of robot motion. Importantly, the system is controlled and programmed by humans, as the working scenarios and task objectives are based on human adaptive intelligence. This flexibility allows for seamless human–robot interaction, effectively combining visualization with control for multitasking objectives.

### 2.4. Innovation of the System

In contrast to previous research utilizing VR for HRC, our study stands out by addressing diverse work requirements across different scenarios. Unlike systems designed for specific industrial settings or focused solely on remote control and display challenges, our system integrates functionalities from prior VR-based HRC research. The goal is to maximize the advantages of VR technology in response to varied working conditions. The system achieves this by incorporating preset packages and streamlined menus, reducing the complexity of information retrieval across different platforms. It seamlessly connects the entire workflow, spanning from the user to VR devices, computers, robots, and real-world work environments. In essence, our system represents a novel approach that leverages advanced data communication, system design frameworks, and user interfaces to fully exploit the benefits of VR technology in the field of robotics. This distinguishes our research from previous studies focused on VR-based HRC.

### 2.5. Advantage of the System

In contrast to previous VR applications in HRC, which often suffered from limited application scenarios and underutilization of VR devices, our system addresses the shortcomings of existing VR-based robot interface research. Most studies primarily focused on using VR device controllers, headsets, and other peripherals for human–machine interaction, limiting the scope of operations compared to our comprehensive approach. Our research builds upon existing VR applications in HRC to overcome challenges such as poor adaptability to diverse scenarios, narrow task execution, insufficient human–machine interaction, and inadequate visualization of information. By doing so, our system contributes to advancing the capabilities of VR technology in the realm of robotics, providing a more versatile and immersive human–robot interaction experience across various scenarios.

## 3. Design and Framework for the System

### 3.1. Concept of the Base Framework

To further harness the potential of VR in the field of HRC, we have integrated five key features of VR, leading us to propose the concept of a VR Human–Robot Five-Dimensional Collaboration System (VR-HR-FDCS), as illustrated in Equation (1):

(1) $$S_{VR}=(CT,\;Vi,\;IA,\;UA,\;FA)$$

In this equation, *S_VR_* represents our system, *CT* represents Control, *Vi* represents Visualization, *IA* stands for Interaction, *UA* represents Usability, and *FA* signifies Infrastructure. The key aspect of the VR-HR-FDCS lies in the integration of middleware and data-to-communication protocols for facilitating communication across various terminals and devices.

**Figure 1:**
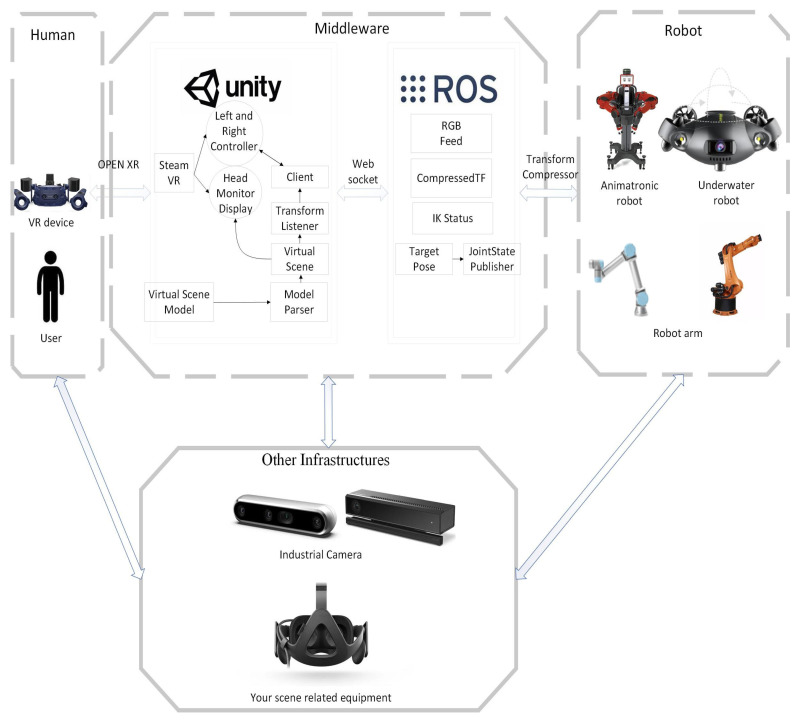
System hardware and software framework.

With each level, data are organized by independent protocols corresponding to the middleware and devices. The system efficiently transfers raw data from certain devices to the middleware, such as constructing scenes within VR devices based on the geometric and physical information of real robots, and subsequently controlling the position of industrial robot end-effectors. On the other hand, some middleware employs publisher–subscriber communication protocols to collect data from devices. These communication protocols are capable of converting data from different terminals and platforms into information that both human operators and middleware can access. Furthermore, the middleware can organize and store these data systematically, providing opportunities for information reuse and retrieval, thus offering the requested information. The concept of this system emphasizes the provision of information with distinct purposes and formats from various terminals, enabling seamless collaboration between humans and robots. Figure 1 illustrates the hardware and software framework of the system.

The research for this system is accomplished by utilizing the Unity engine and ROS platform as middleware. It establishes a connection between VR devices and the Unity platform through Open XR, while also linking the ROS platform with the physical robot. Subsequently, it bridges the Unity engine and ROS platform, enabling the connection of VR devices to the virtual scenes within the Unity platform. The engine communicates with the ROS platform through the publication and subscription of topics, facilitating connectivity with the operating system of the physical robot. Within the virtual robot’s system, commands are input to control the devices in the virtual scene of the engine. These commands are then converted into data formats that ROS can receive and process, allowing for their execution. Furthermore, beyond the foundational components of the system, additional devices such as industrial cameras, depth-sensing cameras, and wireless controllers can seamlessly integrate into the system’s framework by connecting to computers or VR devices.

### 3.2. Communication between ROS and Unity Middleware and Devices

Both ROS and Unity serve as middleware in the system. However, they operate on different computer platforms. ROS is developed for Ubuntu/Linux systems, and its communication with the robotic arm is relatively straightforward. By connecting ROS to the robotic arm via an Ethernet cable and synchronizing the IP settings between ROS’s operating system and the robotic arm, they can establish a connection. This allows data to flow between the ROS and the physical robotic arm. Consequently, the computer client system can send and receive topics published by ROS, enabling ROS to send corresponding commands or data to control the physical robotic arm.

In contrast, Unity development often takes place on Windows systems, which can lead to incompatibility between the two platforms and prevent direct data exchange. Therefore, in the development of this system, a fundamental networking feature called the Socket protocol is employed. The Socket protocol is a full-duplex communication protocol used to facilitate data interaction between clients and servers. With just one connection, a client can establish a long-lasting connection with a server and engage in bidirectional data transfer.

**Figure 2:**
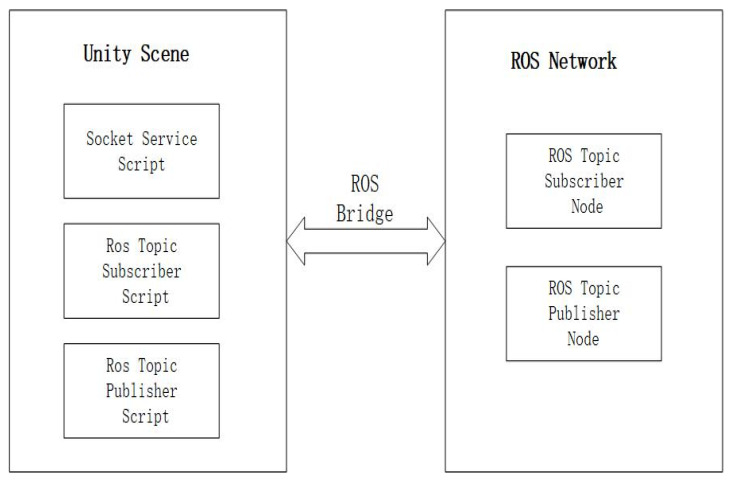
Unity–ROS communication method.

ROS provides the Rosbridge_suite package, and the Rosbridge_server package, which is responsible for managing communication transport layers, offers support for communication schemes such as Socket, TCP, and UDP. Thus, in this study, the Rosbridge_server sub package is utilized to establish connectivity between Unity and ROS. The communication methods between the two ends are depicted in Figure 2.

As depicted in Figure 2, in Unity, scripts are written to invoke the Socket API for establishing a network connection. This involves creating a Socket object to represent the endpoints of the connection, which are comprised of ROS’s IP address and port number. Once ROS binds its Socket object to its IP address and port number, Unity sends a connection request. Upon receiving and accepting this request, the system generates a new Socket object to represent the connection with Unity.

Once the connection is established, both parties engage in data transmission and reception, as well as data interaction, over the new Socket object. Since Unity primarily employs the C# programming language, encoding is required to convert the transmitted information into the format of ROS topics for seamless communication between the two systems.

The coordinate axes for the robot’s base differ between the Unity and ROS environments. It is worth noting that Unity uses a left-handed coordinate system with the *Y*-axis pointing upwards, whereas ROS utilizes a right-handed coordinate system with the *Z*-axis pointing upwards as the reference in its world coordinate system. Consequently, for a seamless connection between the two systems, coordinate system transformation is necessary, as illustrated in the following equations:

(2) $$U_{X}=R_{-Y}$$

(3) $$U_{Y}=R_{Z}$$

(4) $$U_{Z}=R_{X}$$

(5) $$U_{Rotation}={-R}_{Rotation}$$

**Figure 3:**
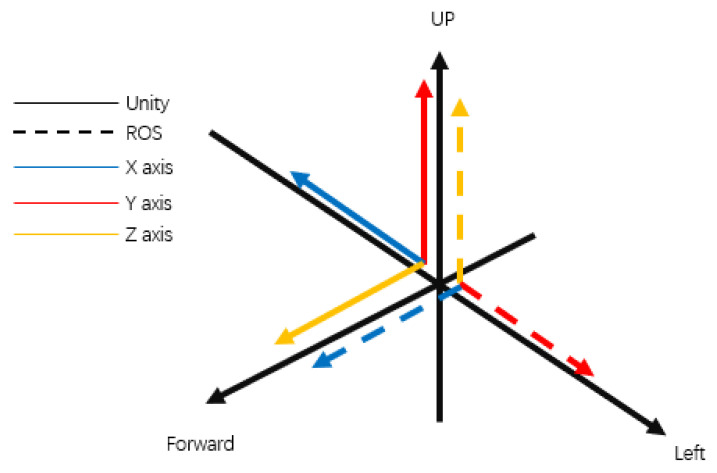
Coordinate conversion between Unity and ROS.

In Unity, the axes correspond to the front (*Z*-axis), right (*X*-axis), and up (*Y*-axis) directions, with rotations occurring clockwise. In ROS, the axes are aligned differently: *X*-, *Y*-, and *Z*-axes, with rotations taking place counterclockwise. To facilitate seamless communication, coordinate transformations are applied during data transmission between the two platforms. This ensures that the models remain compatible and synchronized in their movements. The transformation of its coordinate system is different as shown in Figure 3.

### 3.3. Systematic Data Connectivity

In the VR-HR-FDCS, data formats between various components of the framework are often complex and incompatible. Connecting the virtual and physical environments requires geometric and physical modeling of the real-world environment and devices. When dealing with the physical model of the robot, there are intricate interdependencies and hierarchical relationships among joints and linkages. Behavior constraints need to be applied as well. While the ROS provides official model packages for major robot brands in Unified Robot Description Format (URDF) format, this format is not compatible with Windows-based systems. To ensure that the geometric and kinematic models of the virtual robot match the actual model accurately, without omissions or errors, this study focuses on converting the robot URDF files provided by ROS into Unity’s component format. In Unity, these files are accepted and transformed into component format. Additionally, forward kinematics components are added to the virtual robot model. This enables the visualization of the end effector’s position by controlling the virtual robot’s movements. A comparison is then made between the virtual robot’s motion and the simulated robot’s motion in ROS to verify the correctness of the model’s structure. Furthermore, other objects in the virtual scene, such as assembly targets and components, are defined to make the scene closely resemble the real environment. Once the VR devices are connected, Unity is launched, and the SteamVR software (with automatic updates) is started. This allows users to set up the devices and connect them to the virtual scene. When the devices, including the positional trackers, headsets, and controllers, are configured, Unity displays the scene on the head-mounted display of the VR device. The device controllers’ positions and operation instructions are tracked in real-time by Unity to accept user motion control commands.

The received command information is then encapsulated into the ROS’s information format and transmitted to ROS through a Socket connection. Software packages within the system are written to listen for the sent information and relay it to the ROS MoveIt control software. This, in turn, controls and displays the real mechanical arm’s motion information in RViz. Importantly, this process is reversible, meaning that information from the actual mechanical arm can be read and sent to Unity, allowing the real mechanical arm’s motion information to be displayed in the VR device. 

## 4. System Principles and Case Validation

In the framework of the VR-HR-FDCS, the control of the robot to accurately complete assembly tasks are achieved by monitoring the position of a person’s hands and button commands. VR devices are chosen as the primary tools for capturing user input. Data obtained from these devices undergoes Kalman filtering before being sent to the ROS. To enhance control smoothness and fluidity, an interpolation process is considered. This process interpolates between the current device position and the target position calculated using a Proportional-Integral-Derivative (PID) controller. Linear interpolation is applied to position values between the two poses, and the interpolated positions are executed sequentially. This approach generates smoother trajectories for the robot’s motion and produces precise control signals, facilitating real-time and accurate interaction between the user and the robot.

### 4.1. Five-Dimensional Characterization of the System

#### 4.1.1. Control

**Figure 4:**
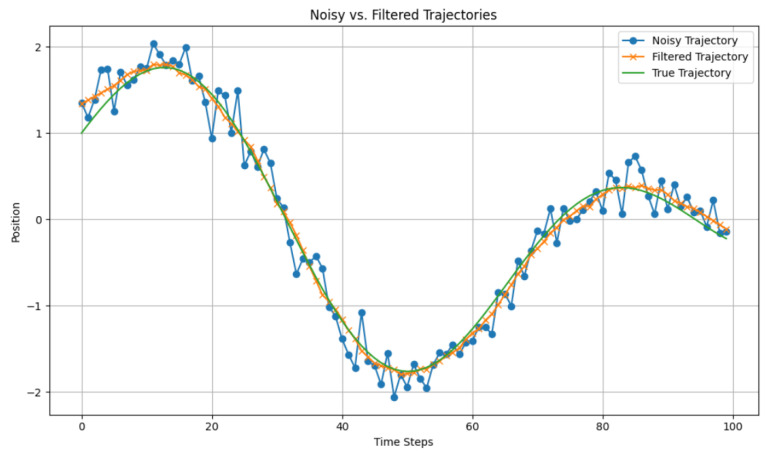
Trajectories before and after filter processing.

The VR device comprises a head-mounted display, left- and right-hand controllers, two infrared emitter locators, and data connection cables. Upon activation of the device, the locators start emitting invisible infrared light signals through their built-in infrared emitters, projecting these signals throughout the usage space. The display and controllers are powered on, and the device’s integrated infrared receivers pick up the emitted infrared light, facilitating spatial positioning of the infrared devices. To ensure accurate localization, the locators control the blinking of the infrared light, maintaining strict time intervals between each emission. The positions of sensors installed on each device are pre-calibrated and fixed, allowing the calculation of the position and motion trajectory of each device based on the differences in sensor positions. In this system, the position of the robot’s end effector is controlled using the position information from the VR device’s controllers rather than controlling individual joint angles. This method is better suited for industrial assembly and manufacturing processes. The controllers provide both position information and button command data. Notably, the degree of button press can offer more precise control. Therefore, the position information from the controllers is obtained within the Unity scene. However, users tend to introduce unintentional jitter or offset in position, even when not in motion. This jitter affects control accuracy. To mitigate this effect, Kalman filtering is applied to the position information from the controllers, resulting in smoothed trajectory information, as shown in Figure 4. The trajectory information is then transmitted through the system to ROS. Upon monitoring the motion information, ROS utilizes MoveIt_Commander to control the robot’s motion.

#### 4.1.2. Visualization

**Figure 5:**
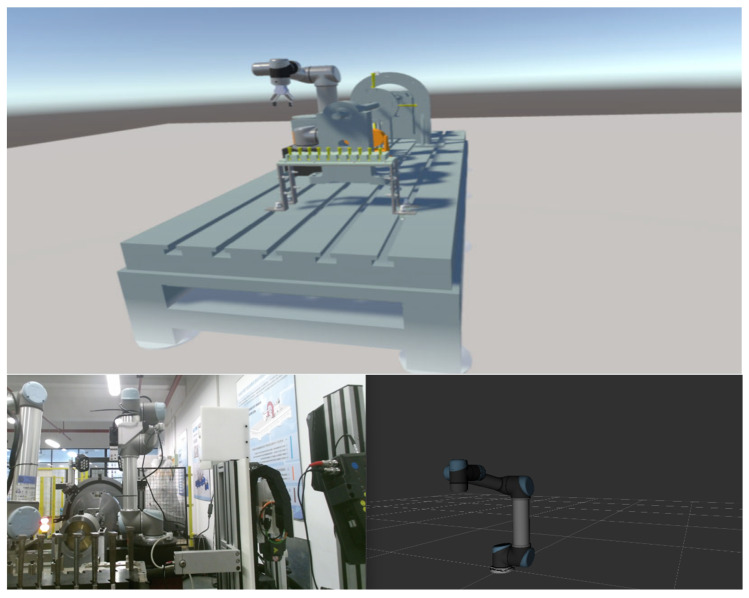
Visualization of the system: Unity and virtual scene.

The visualization module is primarily centered around the VR robot interface. Unlike traditional 2D display surfaces, VR devices provide an immersive virtual environment and scene where we can directly observe the robot’s motion, as if we were physically present on-site. Moreover, the digital capabilities within the virtual scene allow us to provide feedback on the robot’s actual movements. This enables us to clearly visualize the robot’s motion trajectory, joint angles during movement, end effector positions, and other essential information. As a result, even in remote environments, we can disregard distance limitations, providing us with a more intuitive means of reading and accessing real-time information about the actual robotic arm’s movements. Therefore, during the system’s development, we aimed to allow Unity to capture and display the visuals and information from ROS’s RViz software. To achieve this, we utilized an open-source ROS visualization project called Webviz. This project enables us to access RViz’s visuals on the web and, in turn, use a WebView component in Unity for embedding web content within the virtual scene. As a result, we can display RViz’s simulation visuals within the Unity project. Furthermore, the system provides an interface for connecting to cameras on the robot. This interface allows us to display live camera feeds within the virtual scene. Consequently, we can simultaneously observe the actual and simulated robot movements in the virtual environment, along with various data information during motion. Combined with our actual experimental scenarios, the visualization that our system can achieve is shown in Figure 5.

#### 4.1.3. Interaction

The VR system incorporates a variety of devices and middleware that provide diverse pose- and motion-control information. In addition to using the position of the controllers as a control signal, we can also implement various quick and straightforward functionalities through the buttons on the controllers. Each controller has multiple buttons, a trigger button that can detect the extent of the press, as well as touch-sensitive and clickable touchpads. This array of control methods enriches the system’s interactive capabilities and potential applications. Therefore, we have configured the logic for controlling the hand controller’s position information to activate when a designated application control button is pressed. This enables us to start recording the controller’s motion information and convert it into motion control signals. Furthermore, the virtual scene layout in the Unity game engine allows us to make the motion logic of various components in our virtual scene closely resemble real-world scenarios. By setting up inverse kinematics (IK) motion for the robot within the virtual scene, we ensure that the virtual robot behaves with the same motion logic as the actual robot. This means that we can perform tasks within the virtual scene that mirror those in the real-world scenario. We also mark potentially hazardous areas within the real-world scene in the virtual scene. However, we do not restrict the operator from accessing these areas. This approach allows us to intuitively inspect any issues that may arise during movements and mishaps in certain areas, thanks to the offline simulation effect. Distinguishing itself from typical HRC scenarios with single control mechanisms and conventional data displays on single screens, the VR-HR-FDCS offers a more diverse range of interactive operations and intuitive data visualization. It seamlessly combines with previous frameworks, enabling us to provide convenient and efficient control and integration for common operations or displays through the system’s interactivity. Furthermore, it facilitates the development of additional functionalities on top of the existing system. The development of the system is primarily focused on common functionalities such as motion control, data visualization, and monitoring.

#### 4.1.4. Usability

Traditional methods for controlling robot motion often come with a steep learning curve. Whether using a robot teach pendant and controller directly or programming the robot through PLCs and software, operators typically require substantial experience and spend a significant amount of time familiarizing themselves with the environment and operations. Additionally, different operating systems on various platforms are often incompatible, and the displays may not be intuitive. This can result in many of the specialized functionalities of the system not being fully utilized. Given this context, our system aims to make the control interface visual and user-friendly. We achieve this by incorporating menus and tooltips, streamlining the user interface. While retaining functionalities related to replicating physical scenarios and meeting specific debugging and simulation needs, we simplify other operations and features. By doing so, we reduce the complexity and entry barriers for operators. They only need to become familiar with a few buttons and the corresponding functions within the interface, enabling them to quickly access the virtual environment for offline scenario simulation and direct control of actual robots.

**Figure 6:**
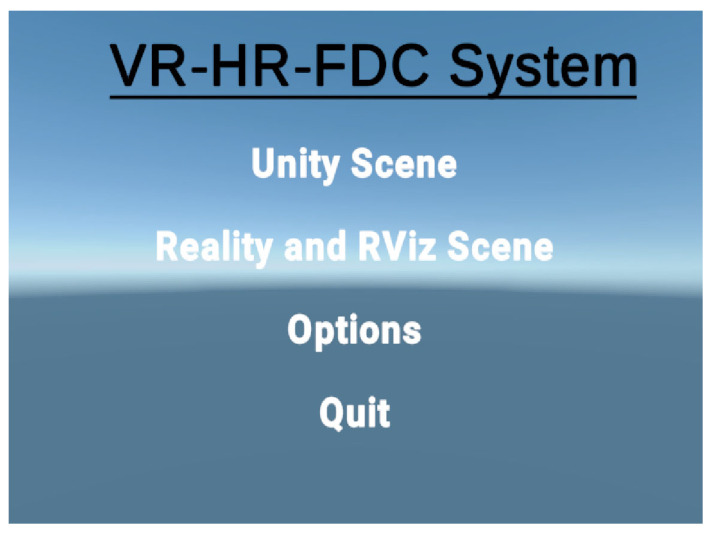
The system’s interface menu.

The clean and user-friendly interface of the virtual platform enhances the user experience. Whether an operator is a robotics expert or a beginner, they can quickly adapt to the system. This approach proves highly effective for comprehensive learning about robots and controlling their movements. The lower entry barriers increase the system’s usability and overall efficiency, leading to a significant improvement in utilization rates. For general operational efficiency in real-world scenarios, simplicity with fewer but well-crafted options is a key consideration in most system interface developments. In line with the distinctive features and usability of our system, we optimized menu options, preserving the display of different scenarios’ characteristics. Options such as changing control methods and interactive settings for different scenes are placed in the settings menu. This ensures the main menu of our system remains concise and intuitive, reducing the impact of users’ familiarity with the system. Therefore, our main menu only includes two visual scenes, settings, and an option to exit the system. The system menu we designed is illustrated in Figure 6.

We have straightforwardly placed two scenes under distinct options. Once the system is connected, within the VR headset, the menu interface of the Unity engine is visible. Through the controller’s button interactions, users can swiftly navigate to the system’s scene interface. This is particularly applicable to our two primary scenes, namely Unity and the real-world RViz scene, allowing for varied visualization and control requirements. Moreover, in different scenes, users can press the menu button on the controller to switch scenes, access settings, or exit scenes, facilitating easy selection of other options.

#### 4.1.5. Infrastructure

Infrastructure-focused elements in this system are aimed at facilitating the development of interfaces connecting VR and robots through system architecture or software. As mentioned earlier, most research leverages ROS for robot development and Unity for VR development. It is essential to note that this system is not a closed ecosystem where components and devices cannot be modified. Instead, the system adopts robust and efficient communication protocols to ensure a linear flow of communication interfaces and data within the system. Operators can transmit models from different scenarios and various control command information through the system’s default framework while maintaining accurate and effective communication. Even with different robot models or various application scenarios and tasks, the system offers excellent compatibility for operators to develop and configure. This significantly reduces the learning curve for operators on the robot system platform.

#### 4.1.6. Five-Dimensional Integration and Mechanisms of the System

The system relies on fundamental hardware components, namely VR devices and computers. It integrates these components by developing scenes and functionalities in the Unity engine, connecting them with the ROS to seamlessly amalgamate the system’s five-dimensional features. Post-connection, control functionalities are realized by pressing VR device controller buttons and manipulating the controllers. The VR device’s head-mounted display furnishes a visual scene, encompassing both the developed virtual environment and the simulation interface from the ROS. Interaction occurs through moving the VR device controllers or pressing buttons, introducing new interactive possibilities based on user gaze from the headset.

Usability is manifested in the system’s streamlined operational interface developed in the Unity engine. It provides simple controls and interactions through buttons, enabling users to quickly familiarize themselves with the system. Additionally, the system’s scalability is evident in the connection between the Unity engine and ROS on the computer. The Unity engine serves as a robust platform, facilitating connections between different scenes and devices. Its compatibility and powerful compilation capabilities, including binding functionalities to controller buttons and compiling interaction modules for diverse devices in various scenes, allow the system to respond to distinct operational needs and industrial settings. This scalability extends to compatibility with other software, contingent on a foundational understanding of Unity. The ROS, as a venerable robotic operating system, accommodates different robot brands, requiring only the replacement of robot program packages to adapt the system to varying operational scenarios and settings.

#### 4.1.7. Systematic Adaptation and Learning Mechanisms

The system’s adaptability to diverse industrial settings and various robots stems from its openness. The Unity engine’s support for different scene models and its inherent compilability enables the system to accommodate various industrial environments. To simplify the operator’s modeling process, additional device models are strategically placed in the system’s default scenes. Leveraging the ROS’s capability to obtain software compilation packages for robots, we transform and integrate models from different robots into the Unity engine’s development scenes. Notably, we have precompiled models for common industrial robots such as KUKA, FANUC, ABB, YASKAWA, etc., within Unity’s extension package. Users can conveniently select the robot model they use, adapting it to their real working scenarios. The system’s five-dimensional features are compatible and applicable in this context.

Certainly, in contrast to the adaptive learning method proposed by Zhao and Lv [39], which aims to achieve output-feedback robust tracking control for systems with uncertain dynamics using techniques developed for optimal control, and Liu et al. [40], who pioneered the application of evolutionary theory to graph structure learning, our system’s learning mechanism primarily focuses on the operators’ self-directed learning. Specifically, it emphasizes the system’s usability. This approach involves simplifying the operational interface, providing textual explanations for various functions, and annotating the interface to expedite operators’ familiarity with system operations. The learning process involves understanding the system’s adaptability across different scenarios, and these efforts are predominantly reflected in the system’s scene development and our instructional materials.

### 4.2. Scenario Application of the System

**Figure 7:**
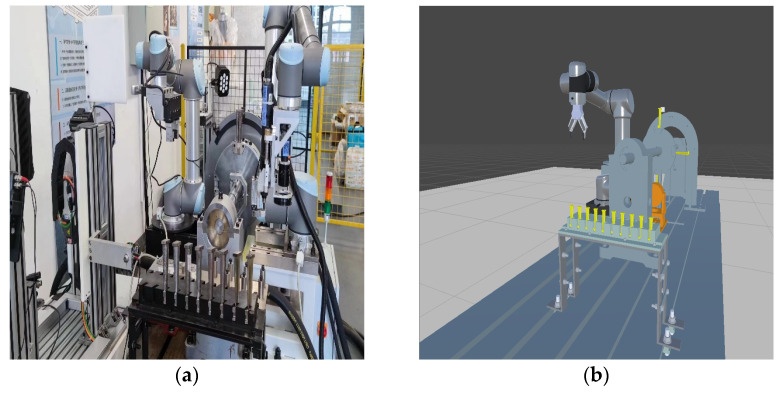
Scene composition of the system: (**a**) real scene; (**b**) virtual scene.

This study presents an intelligent assembly system based on a blade-rotor testbed [41], as shown in Figure 7. The system primarily consists of a UR5 collaborative robotic arm, a servo motor-controlled rotor testbed, and a material rack for holding the components to be assembled. The rotor testbed comprises a servo motor, a rotating axis, an oil-film lubricated rotor, and a turbine blade. The UR5 collaborative robot arm is equipped with an RG6 electric gripper, serving as the end-effector for grasping components. Additionally, a depth camera is mounted at the arm’s end for object recognition and localization. The robotic arm is connected to the main system via Ethernet. ROS is utilized to receive and process signals from various sensors, generate corresponding control signals, and command the robot to perform the required actions.

**Figure 8:**
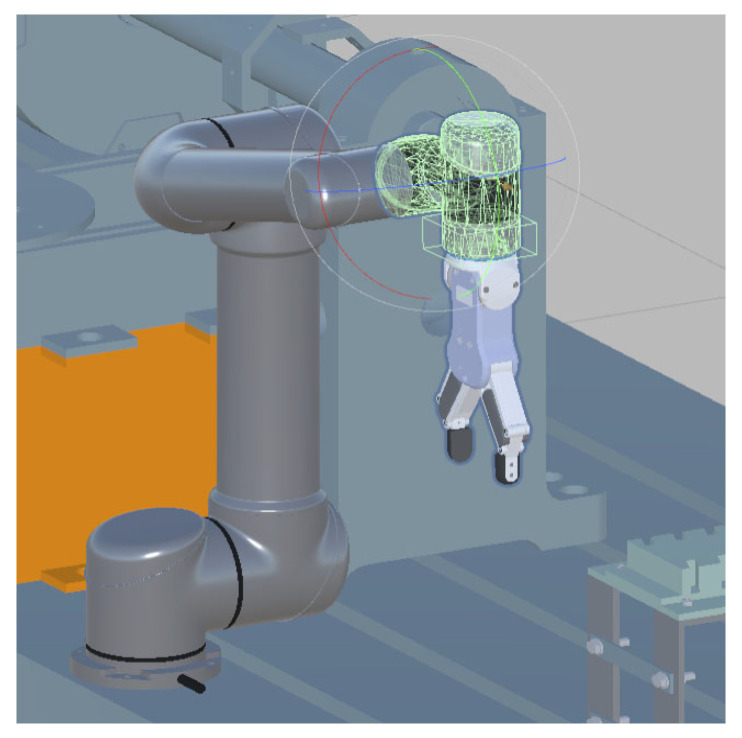
Virtual model hierarchy for UR5 robotic arm: model joint set, red line: *X* axis, blue line: *Z* axis, and green line: *Y* axis.

By conducting a structural analysis of the entire system, a corresponding virtual scene of the physical experimental system was created. Each device and material tool in the scene was individually modeled and constructed. The primary device, the robotic arm, has a complex model, including geometric models and hierarchical relationships between its joints. To achieve this, the URDF file of the official robotic arm model was converted, allowing for the creation of the robotic arm’s scene in Unity, as shown in Figure 8. Additionally, inverse kinematics (IK) motion binding was applied to the robotic arm’s joints, resulting in a virtual scene model that closely resembles the real robotic arm and its environment.

After setting up the virtual scene, the HTC VIVE device was connected to the computer, and the robotic arms of the testbed were started. The computer with the ROS was connected via TCP/IP protocol to initiate the RVIZ software for the visual control and observation of the robotic arm’s movements.

**Figure 9:**
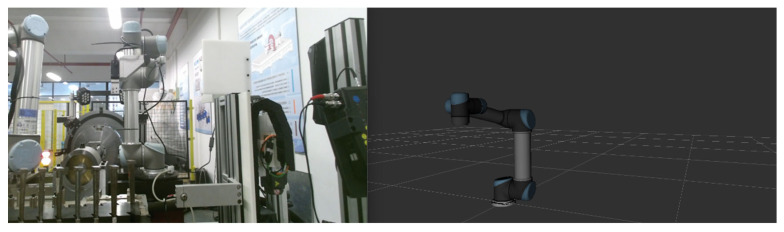
Imaginary work screen of the robotic arm.

Beyond fundamental interaction and control, our system introduces innovation in its visual interface by integrating images of the robotic arm’s movement from RVIZ with the industrial camera on the test bench. RVIZ provides a real-time display of the robotic arm’s motion trajectory and status, while the industrial camera offers an intuitive representation of the actual robot working environment. The combination of these two sources enables a more intuitive observation of the virtual and real movement of the robotic arm. These scenes are manifested within our VR device, effectively presenting the fusion of both perspectives. The VR device adeptly combines these visuals, and the real-time control facilitated by the handheld devices allows us to dynamically observe, adjust, and supervise the robotic arm’s movements. This innovative visual interface significantly enhances overall monitoring and adjustment capabilities within our system. The supervision of the system’s movements could be achieved by accessing both the industrial camera and the RVIZ page, as shown in Figure 9.

By observing the basic blade-grasping task, the system demonstrated its ability to effectively achieve its intended tasks. Combining this with the RVIZ interface allowed for real-time monitoring and observation of the robot’s work status and scene, helping to detect any motion deviations that may occur during the operation.

The blade grasping operation has been divided into six sequential steps, labeled as Q1, Q2, Q3, Q4, Q5, and Q6. Control and display information for each of these steps is documented in Table 1.

**Figure 10:**
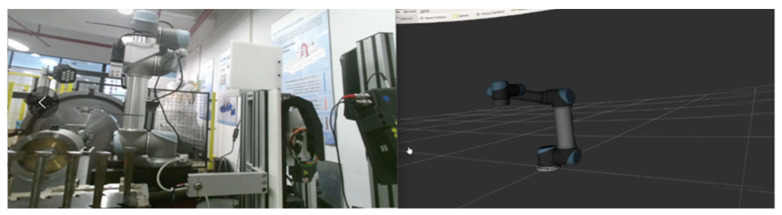
Q1 reality and RViz scene interface.

The initial state of the robot arm is shown in Q1, and the HRC reality and RViz scene interface in the VR device are shown in Figure 10.

**Figure 11:**
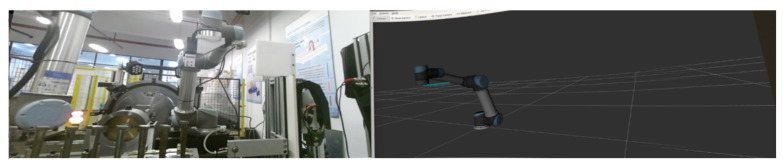
Q2 reality and RViz scene interface, observe the position of the end of the robot arm.

The robot arm in Q2 extends forward and hovers directly above the target blade; the HRC reality and RViz scene interface in the VR device are shown in Figure 11.

**Figure 12:**
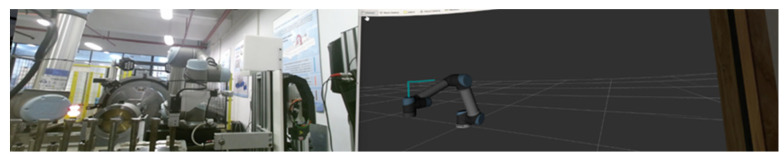
Q3 reality and RViz scene interface.

The robot arm in Q3 descends to the position of the target blade and clamps the target blade; the HRC reality and RViz scene interface in the VR device are shown in Figure 12.

**Figure 13:**
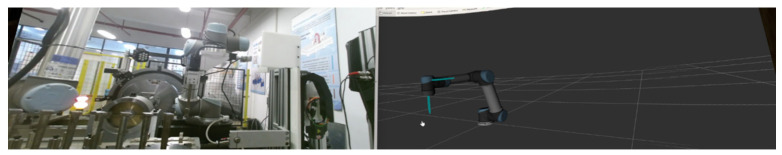
Q4 reality and RViz scene interface.

In Q4, the robot arm clamps the target blade and then rises for a distance, at which time the clamping jaws of the robotic arm clamp the blade; the HRC reality and RViz scene interface in the VR device are shown in Figure 13.

**Figure 14:**
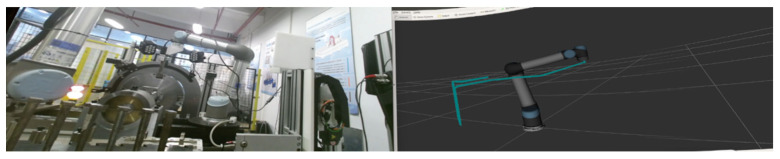
Q5 reality and RViz scene interface.

In Q5, the robot arm places the clamped target blade on the carousel for assembly; the HRC reality and RViz scene interface in the VR device are shown in Figure 14.

**Figure 15:**
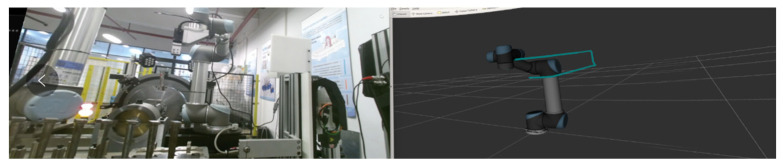
Q6 reality and RViz scene interface.

In Q6, the robot arm is reset and ready for the next clamping task. At this point, the blades have been assembled on the turntable, and the HRC reality and RViz scene interface in the VR device are shown in Figure 15.

**Figure 16:**
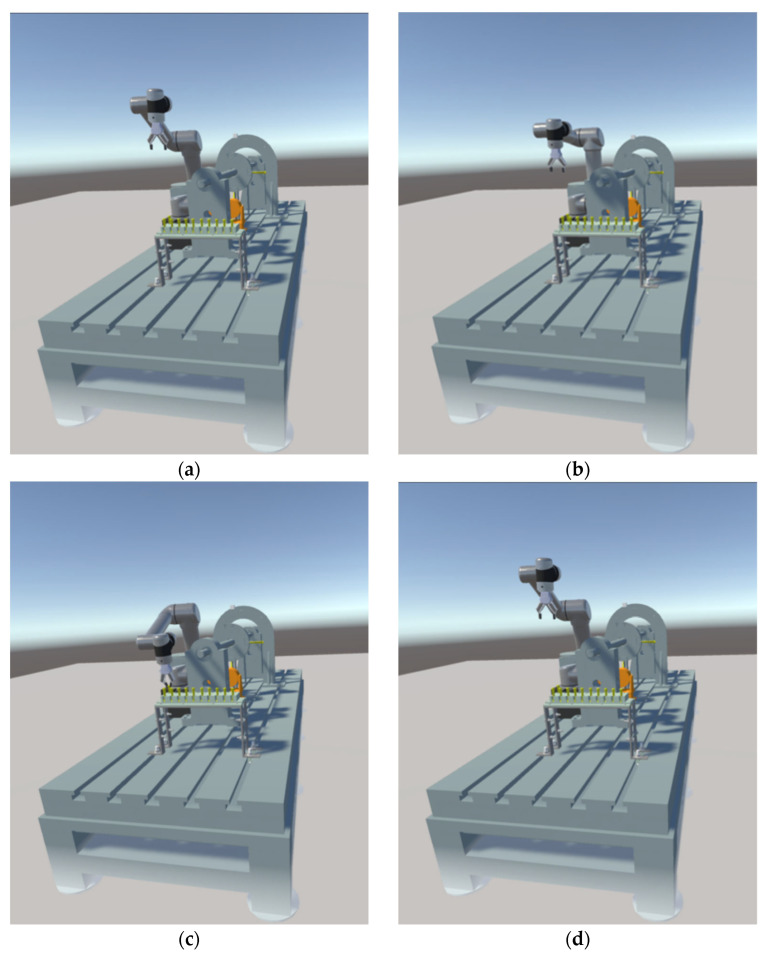

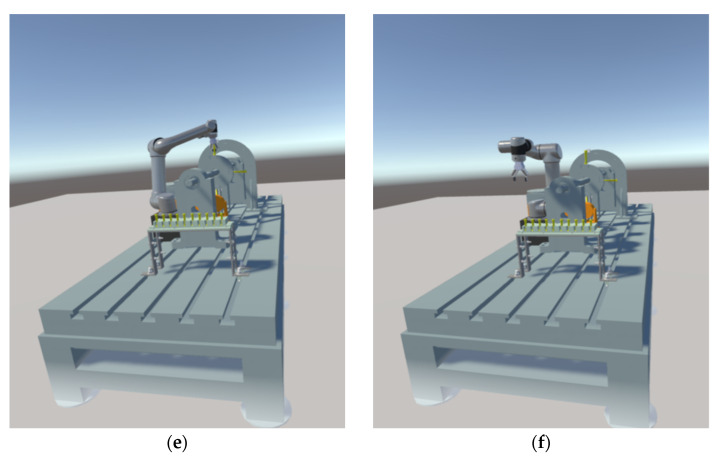
Q1 to Q6 Unity Scene Interface: (**a**) Q1; (**b**) Q2; (**c**) Q3; (**d**) Q4; (**e**) Q5; (**f**) Q6.

We show the realistic and RViz screens in VR devices in the above series of figures, while the Unity screens from Q1 to Q6 are displayed in VR as shown in Figure 16.

Combined with Figure 10, Figure 11, Figure 12, Figure 13, Figure 14, Figure 15 and Figure 16, testing of the VR system was conducted in a room outside of the test bed. The effectiveness of the system’s five-dimensional capabilities was validated through gripping and assembly operations on the blades placed on the test rig. The system demonstrated excellent compatibility with the models of various devices within the scene. It seamlessly connected and operated in tandem with the industrial cameras situated on the test bed, ensuring stable performance throughout the testing process.

## 5. Analysis

### 5.1. Analysis of Experimental Results

Upon powering up the equipment and initiating the entire system, we are presented with a clear view of the virtual scene on the display provided by the VR device. This scene faithfully replicates the real-world environment and allows for basic interactions with the objects therein, delivering feedback akin to that experienced in the actual physical environment. As such, the system offers an intuitive platform for HRC with enhanced visualization.

Simultaneously, the user interface of the system is straightforward, facilitating the seamless transition between the virtual environment generated by the VR engine and the simulation software within ROS. Through the intuitive displays of the simulation interface, we gain direct insights into the robotic arm’s movements. Furthermore, it provides visual representations of the arm’s initial and subsequent positions, affording us the ability to closely monitor alterations in the position of the robotic arm’s end-effector. Additionally, the system furnishes us with various options, enabling us to observe the positions and angles of other joints as well. Within the system’s control interface, we can initiate and manage the movements of the robotic arm within the simulated environment. This action is accomplished by pressing buttons on the handheld controller. Upon pressing the designated control buttons, the system autonomously captures the current position data from the handheld controller and communicates this information via a communication protocol to the ROS, thereby governing the motion of the robotic arm. This entire process is vividly presented within the VR headset, ensuring an immersive and user-friendly experience. Moreover, the system supports the incorporation of additional models and scenarios, permitting the validation of diverse scenarios within the platform. By converting URDFfiles of different robotic devices and transmitting position data of their respective end-effectors, we seamlessly integrate various components into the system, enabling the execution of a wide range of control operations, Lastly, the system’s user-friendly interface significantly lowers the operational threshold for teleoperation of robots.

### 5.2. User Research Design

We evaluated the system’s operability and compared it with traditional control methods by having individuals with varying levels of experience operate and learn robotic arm movements. This study involved nine participants with different levels of expertise. We initially introduced the system’s features and ensured that participants were familiar with its five dimensions. We provided a brief overview of each system function, and participants performed operations similar to those in the previous experiment, labeled from Q1 to Q6.

To standardize participants’ understanding of the VR system and minimize the impact of Unity Engine and ROS familiarity, we allowed only basic changes in robot categories without the need for scene remodeling and compilation. Participants downloaded other robot model packages in the ROS, and common download commands were placed on the ROS desktop. This allowed users to replace robots in the Unity Engine by extracting and replacing the model package content.

To make targeted comparisons between the VR system and traditional robotic operation methods, participants were categorized based on their proficiency in robot control: three had no prior experience (Participants 1, 2, 3), three were familiar with robot control, including teach pendant control and ROS (Participants 4, 5, 6), and three were highly proficient in robot control, familiar with teach pendant, ROS, PLC, and various controllers (Participants 7, 8, 9). They all had limited exposure to VR technology before the study.

To comprehensively assess the system’s performance and its five dimensions, we designed specific questions combining a standard usability questionnaire with aspects related to the five dimensions. During the evaluation, participants wore VR headsets, operated the robotic arm using controllers for tasks Q1 to Q6 as annotated in the system, and then controlled the computer to change the robot category in the working scene. Afterward, they repeated the tasks with the new robot, and finally, they filled out a survey to provide ratings.

### 5.3. User Evaluation

The results obtained after analyzing the system evaluation for these objects with different experiences are shown in Table 2, with each value totaling five points. In this case, for the problem setup there are partly forward and partly reverse problems; for the scoring of forward problems, we take the average value directly, but for reverse problems, such as problem 3 and problem 8, we take the opposite score as the score for the evaluation of the system.

Our study provided a brief introduction and a short teaching session for the participants. Despite the limited exposure, the results were remarkably positive, as every participant successfully achieved the operational objectives. We integrated the five-dimensional features into ten different questions, enhancing the participants’ specific and comprehensive understanding of these features. This laid a solid foundation for the subsequent comparative evaluations of the system.

The evaluation of the system revealed that participants highly appreciated the system’s intuitive display of robot working scenarios, emphasizing the advantage of visualization through the integration of virtual and real robot conditions. Participants also acknowledged the safety of using the system, as it allowed them to operate and supervise activities in a freely secure environment, removed from actual motion scenes and working conditions. However, ratings for system fluency and control stability were not particularly high. This can be attributed to the participants’ limited experience with VR devices, resulting in unfamiliarity with some basic operating procedures. Practical issues, such as difficulty in switching display screens and inadvertent button presses, were observed during the hands-on experience. These issues underscore the areas for consideration in the future development of the system. Furthermore, the interchangeability of middleware and application scenarios in the system requires a certain level of computer proficiency from the participants. This proficiency includes familiarity with robot control systems and the Unity engine. Notably, participants with some experience in robot operations scored higher in this aspect compared to those with less experience, highlighting the influence of prior robotics experience on system interaction.

In summary, our study successfully demonstrated the effectiveness of the VR system by providing participants with a brief introduction and limited instruction time. The positive outcomes indicate the participants’ ability to smoothly accomplish operational tasks. The integration of five-dimensional features into the assessment questions facilitated a more concrete and thorough understanding of these features. While the system received praise for its intuitive visualization of robot scenarios and perceived safety, challenges related to user experience, system fluency, and control stability were identified, guiding considerations for future system enhancements. Additionally, participants’ varying levels of computer proficiency influenced their interaction with the system, emphasizing the importance of user experience in relation to prior robotics knowledge. Through the utilization and assessment of the VR system, it is evident that operators with varying levels of experience can swiftly acquaint themselves with the system’s diverse functionalities. They can proficiently control the robot and supervise its operational environment. Furthermore, in terms of user experience, participants engaged in the testing process reported that the VR system provided a safer and more comfortable working environment. Experienced operators found the system’s visual interface and the ability to supervise real-virtual work environments particularly appealing, leading them to highly recommend the use of this system. For those with limited or no prior experience, swift familiarity with the system’s operation and the ability to control the robot were achievable. The evaluation tests involving operators with differing levels of experience offer an insightful perspective on the system’s advantages.

Subsequently, similar operations and displays were assessed among the test subjects using traditional control methods, allowing for a comparative examination of the characteristics of the VR five-dimensional system and the advantages it holds over traditional operational approaches. The results obtained are shown in Table 3.

In contrast to the evaluation method employed by Qi et al. [42], our assessment approach involved diverse participants experiencing the system in practical applications. This allowed for the recording and evaluation of the participants’ firsthand experiences. Ratings were assigned to relevant features, and the mean scores were calculated to gauge the participants’ perceptions of the system’s characteristics. This method served as a baseline for subsequent analysis. Through the evaluation of several operators with varying levels of experience, it becomes evident that the VR-HR-FDCS excels in control, visualization, interaction, and usability aspects compared to traditional robot operating methods. However, the addition of infrastructure elements may be preferred by experienced operators since it involves combining software and hardware within the existing system. Nevertheless, the system provides a solid platform and interface that can effectively accommodate other software and hardware components after further learning and use.

In traditional operating methods, inexperienced operators often struggle to directly control the robot and complete tasks, highlighting the system’s compatibility with individuals lacking experience. The results indicate that each inexperienced operator leans toward using this system across these five dimensions. When comparing the execution of equivalent tasks using both methods, the system’s simplified robot control and advantages in remote environments and visual HRC interfaces become evident. These are aspects that are either impossible or highly inefficient in traditional methods. Therefore, evaluating the system based on its five-dimensional characteristics, such as ease of operation and visualization, reveals innovative solutions to the limitations of traditional teleoperation and HRC.

## 6. Conclusions and Prospects

We have developed a VR HRC system based on VR equipment. The VR headset and controllers replace traditional HRC interfaces and input devices. With this system, operators can create, supervise, and execute robotic movements on industrial robots using VR devices. The system offers a simplified, more visually intuitive interface for observing robot motion and assessing the current work environment compared to traditional methods. We conducted a user study involving nine robot operators with varying levels of experience, ranging from novices to experts. The results of the evaluation indicate that even operators with limited prior experience can quickly and efficiently control robots using our system, reducing the workload and operational complexity. The immersive experience provides operators with a better and more intuitive understanding of the real-time robot movements and the work environment. In remote scenarios, robotics experts can supervise real-world work conditions using the system without physically being present. Furthermore, our VR-based HRC system only requires standard commercial VR equipment and a personal computer, making it a cost-effective alternative to specialized teaching devices or highly integrated control consoles found in traditional setups. The system also offers portability and space efficiency, eliminating the need for on-site presence. It is not affected by communication delays or inefficiencies and can potentially replace direct communication with ROS in the future.

Through case analyses of practical scenarios and evaluations conducted by diverse participants, we have identified certain limitations of the system. The deployment of VR devices requires a spatial support of at least 2 m × 1.5 m. While the system can still function in confined spaces, the user’s movement will be significantly restricted. Although teleportation functionality allows for jumping within the VR scene, the overall user experience diminishes. Careful organization of locators and device cables is also crucial to prevent tripping, especially when the user cannot see their actual environment. Additionally, the communication latency between different platforms impacts the control precision and real-time performance of the system. Users’ familiarity with VR devices, Unity engine, and ROS influences the learning time and efficiency of operating the system, especially when changing middleware in different work scenarios and conditions. These challenges represent areas for future system development and improvement.

With the ongoing research and development of VR all-in-one devices by various companies, such as PICO by ByteDance and the upcoming Apple Vision PRO by Apple, the application scope of VR devices is expected to expand. As VR devices become more widespread, simpler to operate, and more powerful, and as engine and operating system compatibility improves, the system’s viability will be maintained. This will allow the system to adapt to different operating and real-world devices in various scenarios and conditions. The continuous development of VR technology and devices, along with the introduction of all-in-one VR devices, will reduce the system’s spatial requirements and minimize latency through optimization of control and communication between different platforms.

## Figures and Tables

**Table 1:** Experimental steps and contents.

Step (Q)	Motion State of the Robot Arm	Operator Operated
Q1	Initial state of the robot arm	Choose to enter the system from the menu
Q2	The robot arm extends forward and hovers directly above the target blade	Hold the trigger button on the controller to initiate control of the robot arm to the designated position
Q3	The robot arm descends to the position of the target blade and clamps the target blade	Hold the trigger button on the controller to reach the position, then press the touchpad button to perform the gripping task
Q4	The robot arm grips the target blade and then rises a short distance	Hold the trigger button on the controller and move the robot arm
Q5	The robot arm places the clamped target blade on the carousel for assembly	Hold the trigger button on the controller, move the robot arm, and press the touchpad button upon reaching the position to perform the placement task
Q6	The assembly is completed, the arm is reset and ready for the next clamping task	Press the grip button on the side to initiate the resetting operation

**Table 2:** Functional evaluation of VR systems by testers.

Question	1	2	3	4	5	6	7	8	9	Mean	Positive Value
1. I find the operation of the system simple and I can familiarize myself with it quickly	5	5	4	4	5	3	4	3	5	4.22	4.22
2. I can perform the operations I need to perform smoothly	5	4	4	4	5	4	4	3	4	4.11	4.11
3. I think the control stability of the system is poor	2	1	1	2	2	3	2	1	3	1.89	4.11
4. I think the system’s display of the robot’s working scene is more intuitive	5	5	5	5	5	5	5	4	5	4.89	4.89
5. I think the system’s various function modules fulfill the basic operation requirements	5	4	5	5	5	4	4	4	5	4.56	4.56
6. I think the system can work well in a remote environment	4	5	4	5	3	5	5	4	4	4.33	4.33
7. I feel that the system is safe to use in a safe environment	5	4	5	5	5	4	5	5	5	4.78	4.78
8. I feel that the system is not very comfortable to use	1	2	1	1	1	2	2	3	2	1.67	4.33
9. I can easily change the scenarios of the system	3	4	4	3	4	4	4	4	5	3.89	3.89
10. I also prefer to use VR systems in general	4	4	5	5	4	5	4	3	3	4.11	4.11

Note: The total score is a five-point scale, with a minimum of one point. Non-positive evaluation scores are calculated by taking the corresponding positive score.

**Table 3:** Tester’s assessment of the VR system compared to traditional methods.

Dimension	1	2	3	4	5	6	7	8	9	Percentage
1. Control	O	O	O	O	O	O	X	O	X	77.78%
2. Visualization	O	O	O	O	O	O	O	X	O	88.89%
3. Interaction	O	O	O	O	O	O	O	O	O	100.00%
4. Usability	O	O	O	O	X	O	O	X	O	77.78%
5. Infrastructure	O	O	O	O	X	O	X	X	O	66.67%

Explanation: Operators who feel that the VR system is better hit O, and those who feel that the traditional way of operation is better hit X. The formula for calculating the percentage is $\frac{O}{\left(O\;+\;X\right)}\times 100\%.$

## Data Availability

No underlying data were collected or produced in this study.
